# Wu-Mei-Wan Ameliorates Murine Ulcerative Colitis by Regulating Macrophage Polarization

**DOI:** 10.3389/fphar.2022.859167

**Published:** 2022-03-21

**Authors:** Shuguang Yan, Hailiang Wei, Rui Jia, Meijia Zhen, Shengchuan Bao, Wenba Wang, Fanrong Liu, Jingtao Li

**Affiliations:** ^1^ College of Basic Medicine, Shaanxi University of Chinese Medicine, Xianyang, China; ^2^ Key Laboratory of Gastrointestinal Diseases and Prescriptions in Shaanxi Province, Shaanxi University of Chinese Medicine, Xianyang, China; ^3^ Department of General Surgery, The Affliated Hospital of Shaanxi University of Chinese Medicine, Xianyang, China; ^4^ Department of Gastroenterology, Yulin Hospital of Traditional Chinese Medicine in Shaanxi Province, Yulin, China; ^5^ Departments of Infectious Disease, The Affliated Hospital of Shaanxi University of Chinese Medicine, Xianyang, China

**Keywords:** wumeiwan decoction, ulcerative colitis, MAPK signaling pathways, NF- κB signaling pathway, STAT6 signaling pathway

## Abstract

An increasing body of evidence shows that macrophages play an important role in the pathogenesis of ulcerative colitis (UC). Macrophage polarization and changes in related signaling pathways are reported to have a protective effect on intestinal inflammation. The well-known Chinese medicine Wumeiwan (WMW) has been used to treat diarrhea, one of the main symptoms of colitis, for more than 2,000 years. Increasing evidence shows that WMW can inhibit intestinal inflammation and repair damaged intestinal mucosa, but its effector mechanisms are unknown. Therefore, we studied the prophylactic effects of WMW in dextran sulfate sodium (DSS)-induced UC and its effects on macrophage mechanisms and polarization. The results show that colitis was significantly alleviated in mice in the WMW group, and the secretion and expression of pro-inflammatory factors TNF-*α*, IL-1, and IL-6 were inhibited in the serum and colonic tissues of mice with WMW-treated colitis, whereas anti-inflammatory factors IL-10, Arg-1, and TGF-*β*1 were increased. Subsequent studies found that WMW could inhibit M1 polarization and promote M2 polarization in colonic macrophages in DSS-induced colitis mice. Network pharmacology was used to predict potential targets and pathways, and further studies confirmed the related targets The results showed that WMW gradually inhibits the activation of the P38MAPK and NF-κB signaling pathways and further activates the STAT6 signaling pathway. In summary, WMW interferes with the p38MAPK, NF-κB and STAT6 signaling pathways to regulate M1/M2 polarization in macrophages, thereby protecting mice against DSS-induced colitis.

## Introduction

Ulcerative colitis (UC) is an inflammatory disorder characterized by intestinal inflammation and mucosal injury. The risk of colitis-related cancer increases with disease course (Low et al., 2019). It has been reported that as many as 30% of UC patients develop colorectal cancer (CRC) after 35 years of illness. Therefore, effective prevention and treatment of chronic colitis are of great significance in reducing the incidence of CRC (Rogler, 2014). An important clinical manifestation of colitis is chronic intestinal inflammation. Pathological studies have confirmed the presence of a large amount of immune cell infiltration in the diseased mucosal tissues of UC patients, including macrophages, neutrophils, mast cells, and eosinophils. These cells participate in the development of colitis and the malignant transformation process from UC to CRC by releasing cytokines, chemokines, signaling molecules, and vascular growth factors (Terzić et al., 2010; Ungaro et al., 2017). An increasing volume of evidence shows that macrophages play an important role in the pathogenesis and persistence of UC. It has been reported that macrophage polarization and changes in related signaling pathways have protective effects against intestinal inflammation (De Schepper et al., 2018; Maasfeh et al., 2021). Macrophages can be transformed into classically activated (M1) macrophages or alternatively activated (M2) macrophages according to different environmental stimulus responses. M1 macrophages mainly engulf and kill bacteria, release inflammatory factors, and promote inflammation. M2 macrophages mainly release anti-inflammatory factors, inhibit inflammation, and promote tissue repair (Locati et al., 2020). Clinical studies have proven that dysregulated macrophage M1/M2 polarization is present in colonic lesions in UC patients (Dharmasiri et al., 2021). Therefore, regulating macrophage M1/M2 polarization to achieve M1/M2 balance at the site of inflammatory injury is considered to be an effective method of treatment for UC (Sun et al., 2020).

Wumeiwan (WMW) is made from a famous traditional Chinese medicine formula that has been used to treat diarrhea for two millennia. WMW originates from the “Treatise on Cold Damage Diseases” written by Zhang Zhongjing in the Han dynasty and is used to treat chronic digestive diseases in which diarrhea is the main symptom. Diarrhea is one of the main symptoms of colitis, so WMW is widely used in China to treat UC. An increasing body of evidence shows that WMW has anti-colon necrosis and anti-intestinal fibrosis effects and can inhibit intestinal inflammation and repair damaged intestinal mucosa. However, the specific effector mechanism(s) of WMW in the treatment of UC are not clear (Wu et al., 2020a; Jiang et al., 2020; Wu F. et al., 2021). WMW is made from 10 plants, including smoked plum, wild ginger (Asarum sieboldii), ginger (Zingiber officinale), Aconitum carmichaelii, Zanthoxylum, Cinnamomum cassia, Coptis chinensis, Phellodendron amurense, Panax ginseng, and Angelica sinensis. High-performance liquid chromatography performed on an WMW extraction solution found that the active ingredients of WMW include phellodendrine, berberine, cinnamaldehyde, and 6-gingerol, which may be the material bases for WMW’s effectiveness (Wu et al., 2020b). Recent studies demonstrated that berberine and hesperetin improve intestinal barrier function and inhibit inflammation to ameliorate DSS-induced UC (Zhu et al., 2019; Zhang et al., 2020; Wang et al., 2021). 6-Gingerol was found to regulate the NF-κB signaling pathway to alleviate inflammatory damage in DSS-induced UC mice (Sheng et al., 2020). Cinnamaldehyde was shown to inhibit Th17 cell differentiation and macrophage activation to improve UC (Qu et al., 2019; Qu et al., 2021). Phellamuretin was found to regulate the AMP-activated protein kinase (AMPK)/mammalian target of the rapamycin (mTOR) pathway to promote autophagy and treat UC (Su et al., 2021). Although these study results partially reveal the effector mechanism of WMW in UC treatment, it is still unclear whether WMW and its active ingredients interfere with macrophage polarization to inhibit inflammation and control UC.

Therefore, in this study, we assessed the protective effects of WMW in UC in a DSS-induced colitis model. Network pharmacology was used to predict UC-related targets and pathways, and further experiments validated the effects of WMW on macrophage polarization and related signaling pathways, showing the effector mechanisms of this drug in UC treatment.

## Materials and Methods

### Drugs and Reagents

Dextran sulfate sodium salt (DSS) was obtained from MP Biomedicals. All of the herbs were purchased from Shaanxi Xingshengde Pharmaceutical Co., Ltd. (Xi’an, China). Primary antibodies tumor necrosis factor-*α* (TNF-*α*), interleukin-10 (IL-10), transforming growth factor-*β*1 (TGF-*β*), arginase-1 (Arg-1), and F4/80 were purchased from Affinity Biosciences (OH, United States). Primary antibodies interleukin-1 (IL-1*β*), interleukin-6 (IL-6), extracellular regulated proteinhnase1/2 (Erk1/2), phosphorylated extracellular regulated proteinhnase1/2 (p-Erk1/2), p38mitogen-activated protein kinases (p38MAPK), phosphorylated p38mitogen-activated protein kinases (p-p38MAPK), inhibitor kappa B alpha (IkB*α*), signal transducers and activators of transcription-6 (STAT-6), and phosphorylated nuclear factor-kappa Bp65 (p-NF-kBp65) were purchased from Cell Signaling Technology (Beverly, MA, United States). Primary antibodies phosphorylated inhibitor kappa B alpha (p-IkB*α*), nuclear factor-kappa Bp65 (NF-kBp65), c-jun N-terminal kinase1/2 (JNK1/2), phosphorylated c-jun N-terminal kinase1/2 (p-JNK1/2), and phosphorylated signal transducers and activators of transcription-6 (p-STAT-6) were purchased from Abcam (Cambridge, MA, United States). Primary antibodies CD206 and CD80 were purchased from Santa Cruz (Santa Cruz, CA, United States). APC anti-mouse F4/80 antibody, FITC anti-mouse CD80 antibody, and PE anti-mouse CD206 (MMR) antibody were purchased from Biolegend (San Diego, CA, United States). The secondary antibodies were purchased from Boster (Wuhan, China), and enzyme-linked immunosorbent assay (ELISA) kits for IL-6, IL-1*β*, TNF-*α*, and interferon regulatory factor 5 (IRF-5) were purchased from Elabscience Biotechnology (Wuhan, China). All reagents and chemicals were of analytical grade.

### Medicine Wumeiwan Preparation and Quality Control

The composition of WMW is shown in Table 1. The identification and deposition of the herbs and the preparation of WMW were completed by the College of Pharmacy, Shaanxi University of Chinese Medicine. Briefly, crude herbs were macerated in 80% aqueous ethanol for 2 h and then heated to reflux twice in a water bath (1 h each time). Then, the filtered and mixed suspensions were collected and concentrated. Finally, the extract mixture was vacuum dried for reserve.

**TABLE 1:** Contents of Wumeiwan decoction.

Chinese name	Botanical name	Common name	Genus	Weight,g	Part used
Wumei	Prunus mume	Fructus Mume	Apricot	30	Fruit
Xixin	Asarum sieboldii Miq	Herba Asari	Asarum L.	6	Root and rhizoma
Ganjiang	Zingiber officinale Rosc.	Zingiberis Rhizoma	Zingiber	10	Root
Fuzi	Aconitum carmichaeli Debx.	Aconiti Lateralis Radix Praeparata	Aconitum L.	6	Root
Huajiao	Zantho xylum bungeanum Maxim.	Pericarpium Zanthoxyli	Zanthoxylum L.	4	Peel
Guizhi	Cinnamomum cassia Presl	Ramulus Cinnamomi	Cinnamomum	6	Twig
Huanglian	Coptis chinensis Franch.	Rhizoma Coptidis	Coptis Salisb.	16	Root
Huangbai	Phellodendron chinense Schneid	Cortex Phellodendri	Phellodendron Rupr.	6	Peel
Renshen	Panax ginseng C. A. Mey.	Panax ginseng C. A. Meyer	Panax L.	6	Root
Danggui	Angelica sinensis (Oliv.) Diels	Radix Angelicae Sinensis	Angelica L.	4	Root

HPLC fingerprinting analysis was used to verify the main chemical constituents of the WMW extract. The extracts were dissolved in water at a concentration of 3.6 g/ml (w/v) and further diluted with methanol water (50:50) to 1.8 g/ml (w/v). Samples were separated on an Agilent ZORBAX SB-C18 column (250 mm × 4.6 mm, 5.0 μm) with a mobile phase consisting of A (Methanol)-B (0.1% phosphoric acid) (0.0–120.0 min, 8%–65% A; 120.0–121.0 min, 65%–8% A; 121.0–130 min, 8% A) at a flow rate of 1.0 ml/min with gradient elution. The column temperature was set at 30°C, the injection volume was 10 μL, and the detection wavelength was set to 242 nm.

### Experimental Animals and Grouping

Male C57BL/6 mice (20–23 g, aged 6–8 weeks) were obtained from Changzhou Cavens Laboratory Animals Co., LTD. (Jiangsu, China) with license No. SCXK (SU) 2016–0010. All mice were housed under specific pathogen-free conditions. The mice received humane care in accordance with the Shaanxi University of Chinese Medicine Animal Care Committee guidelines.

To induce acute colitis, mice were given drinking water containing 2.5% DSS for 1 week, followed by normal drinking water for 1 week. The mice were randomly divided into four groups: normal control group (NC, 10 ml/kg, 0.9% saline), model control group (MC, 10 ml/kg, 0.9% saline), and WMW group (WMW, 27 g/kg, 54 g/kg, weight ratio between crude drug and mice). The schematic diagram is presented in Figure 2A. To induce chronic colitis, mice received DSS (2.5% w/v) dissolved in drinking water. The mice were subjected to four cycles of DSS. Each cycle included 1 week of DSS administration followed by 1 week of recovery with normal drinking water (Sun et al., 2020). The mice were randomly divided into three groups: normal control group (NC; 10 ml/kg, 0.9% saline), model control group (MC; 10 ml/kg, 0.9% saline), and WMW group (WMW: 54 g/kg, weight ratio between crude drug and mice). The schematic diagram is presented in Figure 3A.

### Evaluation of Colitis

To evaluate the severity of colitis, body weight, fecal occult blood, and fecal consistency were examined every week to generate a disease activity index (DAI) score (Table 2). All mice were sacrificed, the serum and colon were collected, and the length of the colon was measured.

**TABLE 2:** Disease activity index (DAI) scoring system.

Score	Body weight loss	Fecal consistency	Fecal occult blood
0	0	Negative	Negative
1	1–5%	Soft stool	Light blue
2	5–10%	Mucoid stool	Blue
3	10–20%	Watery stool	Dark Blue
4	>20%	—	Gross blood

To observe morphological and mucus secretion changes in the colon, colon sections were subjected to hematoxylin and eosin (H&E) and periodic acid Schiff (PAS) staining. Samples were dehydrated and embedded in paraffin and sectioned (3 μm) after fixation in a 4% formaldehyde solution for 12 h. Images were collected using an optical microscope. The tissue expression of neutral mucins in goblet cells was determined as a function of PAS staining. Histological scores were examined according to previously studies (Table 3) (Kiyohara et al., 2018).

**TABLE 3:** Histological scoring system.

Score	Severity of inflammation	Depth of injury	Crypt damage	Percentage of the involved area
0	None	None	Negative	0%
1	Slight	Mucosal	Basal 1/3 damaged	1–10%
2	Moderate	Mucosal and submucosal	Basal 2/3 damaged	10–25%
3	Severe	Transmural	Only surface epithelium intact	25–50%
4	—	—	Entire crypt and epithelium lost	50–100%

### Immunohistochemistry Assay

Paraformaldehyde-fixed colon slides were dewaxed and rehydrated according to the standard protocol. Subsequently, endogenous peroxidase activity was blocked with a 3% H2O2 solution after antigen retrieval. Then, the slides were blocked with 10% normal goat serum for 30 min. The sections were incubated with Rabbit anti-F4/80 (1:100) overnight at 4°C and biotinylated secondary antibody (1:100), for 30 min. Boundper oxidase was visualized after a 3,3 diaminobenzidine reaction for 25 min and after light counterstaining with hematoxylin, dehydration, and mounting. Images were collected using a fluorescent microscope (Olympus BX53, Shinjukuku, Tokyo, Japan).

### Immunofluorescence Assay

The 4% paraformaldehyde-fixed colon was cut into 3-µm sections for double immunostaining analysis. The sections were incubated with primary antibody (CD206, CD80, IL-10, IL-1*β*, all at 1:50 dilutions) overnight at 4°C in the dark and FITC-labeled and Cy3-labeled secondary antibody (1:100) for 1 h. DAPI (1:1,000) was used to counterstain the nuclei for 5 min, and the slides were mounted on cover slips with an anti-fade mounting medium. Images were collected using a fluorescent microscope (Olympus BX53, Shinjukuku, Tokyo, Japan).

### Enzyme-Linked ImmunoSorbent Assay Analysis

The mice were anesthetized and sacrificed at the end of the experiment. Serum was obtained after centrifugation at 3,000 rpm for 15 min. Levels of IL-6, IL-1*β*, TNF-*α*, IRF-5 were determined using ELISA kits according to the manufacturer’s protocol.

### Western Blot Analysis

The colon tissue was lysed and homogenized in radio immunoprecipitation assay (RIPA) buffer containing 1% phenylmethylsulfonyl fluoride (PMSF) and phosphatase inhibitors. The homogenate was then lysed on ice for 30 min and centrifuged at 12,000 g for 5 min at 4°C. The protein concentration was determined by the bicinchoninic acid (BCA) method. The proteins (40 μg/lane) were separated by 10% sodium dodecyl sulfate polyacrylamide gel electrophoresis (SDS/PAGE) and transferred to PVDF membranes for 2 h (Millipore, Billerica, MA, United States). After blocking with 5% nonfat milk in TBST for 2 h, the membrane was incubated with primary antibody (IL-1*β*, TNF-*α*,IL-6, IL-10, TGF-*β*, Arg-1, Erk1/2, p38MAPK, p-p38MAPK, IkB*α*, p-IkB*α*, p-NF-kBp65, JNK1/2, STAT-6, p-STAT-6,*β*-actin all at 1:1,000 dilutions; p-Erk1/2, NF-kBp65, p-JNK1/2 all at 1:2000 dilutions) at 4°C overnight. Secondary antibodies against HRP-conjugated rabbit or mouse IgG (dilution 1:5,000, Boster, Wuhan, China) were added for 2 h at 37°C. An enhanced chemiluminescence plus detection system (Glyko Biomedical Limited, United States) was used for detection, and Band Scan software was used to analyze the optical density of the bands.

### RNA Extraction and Quantitative Real Time Polymerase Chain Reaction Analysis

Total RNA from colon tissue was extracted with TRIzol reagent (Ambion, CA, United States). cDNA synthesis was accomplished with HiScript® II Q RT SuperMix for qPCR (VAZYME, Nanjing, China). Quantitative real-time PCR was performed on an ABI QuantStudio 6 (Applied Biosystems, Waltham, MA). Relative mRNA expression was determined by the 2^−ΔΔ^Ct method, with *β*-actin as the internal control. The primer sequences used for PCR are listed in Table 4.

**TABLE 4:** Primer sequence of target genes (All genes species are mouse).

Gene name	Forward	Reverse
IL-1	AGG​CAG​TAT​CAC​TCA​TTG​TGG	ACG​AGG​CTT​TTT​TGT​TGT​TC
IL-6	CAC​AGA​GGA​TAC​CAC​TCC​CAA​CAG​A	ACA​ATC​AGA​ATT​GCC​ATT​GCA​CAA​C
TNF-*α*	GCC​TAT​GTC​TCA​GCC​TCT​TCT	TTG​TGA​GTG​TGA​GGG​TCT​GG
IL-10	ACC​TGG​TAG​AAG​TGA​TGC​CC	ACA​CCT​TGG​TCT​TGG​AGC​TT
TGF-	TTG​CTT​CAG​CTC​CAC​AGA​GA	TGG​TTG​TAG​AGG​GCA​AGG​AC
Arg-1	GGT​AGC​AGA​GAC​CCA​GAA​GA	CAG​CGG​AGT​GTT​GAT​GTC​AG
*β*-actin	CAC​GAT​GGA​GGG​GCC​GGA​CTC​ATC	TAA​AGA​CCT​CTA​TGC​CAA​CAC​AGT

### Flow Cytometry Analysis

Fresh colon tissues were harvested, minced, and then digested with a 0.5% collagenase (Yuanye Bio, Shanghai, China) and 0.25% trypsin solution (Procell, Wuhan, China), filtered, cleaned, and centrifuged to obtain single cells. Single cell suspensions were stained and fluorescently conjugated against mouse CD80, CD206, and F4/80 at the recommended dilutions for 30 min at 4°C in the dark. Each flow tube contained 1 ml of 0.5% bovine serum albumin (BSA) PBS and 1 × 10^6^ cells, Cells were detected using a Beckmancoulter Cyto FLEX and analyzed with FlowJo software.

### Network Pharmacology Analysis

The chemical composition information in WMW was collected from the published literature (Wu et al., 2020a; Jiang et al., 2020; Wu MM. et al., 2021) as well as from the Traditional Chinese Medicine Systems Pharmacology (TCMSP, https://tcmspw.com/tcmsp.php) database. According to the pharmacokinetic parameters of absorption, distribution, metabolism, and excretion (ADME), compounds with oral bioavailability (OB) ≥ 30% and drug likeness (DL) ≥ 0.18 were selected for further research. The potential targets of the abovementioned compounds were collected from the Swiss Target Prediction (http://www.swisst-argetprediction.ch/) website. Targets related to “ulcerative colitis” were found in the pharmGKB database (https://www.pharmgkb.org/), Uniprot database (https://www.uniprot.org/), DisGeNET database (http://www.dis-genet.org/home/), OMIM database (https://www.omim.org/), DrugBank database (https://www.drugbank.ca/), and TTD database (http://db.idrblab.net/ttd/). Information regarding the intersection of WMW and disease targets was obtained from the Venny database (Venny 2.1.0), and the intersection targets were imported into the STRING database (https://string-db.org/) to determine the protein interaction relationship using Cytoscape 3.7.1 software for protein–protein interaction (PPI) analysis. Genes whose degree, betweenness centrality, and closeness centrality were greater than their respective medians were selected as core target genes. The DAVID database (NCIFCrf.gov) was used for the KEGG pathway enrichment analysis. The bubble map of the first 20 channels was generated using the bioinformatics. com.cn website.

### Statistical Analysis

The statistical analysis was performed using GraphPad Prism 7.0 (San Diego, CA, United States). Data are presented as the mean ± SD and were analyzed using an unpaired Student’s t test and a one-way analysis of variance (commonly known as an ANOVA). A *p*-value of <0.05 was considered to indicate statistical significance.

## Results

### Identification of the Chemical Constituents in Medicine Wumeiwan

**FIGURE 1:**
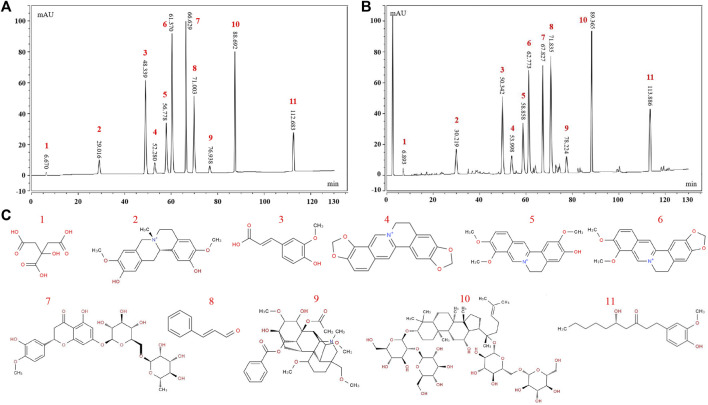
HPLC fngerprinting of WMW. **(A)** HPLC fngerprint chromatograms of thereference standards **(B)** HPLC fngerprint chromatograms of the WMW extracts. **(C)** The structural formula of chemical compounds in WMW: 1 citric acid; 2 phellodendrine; 3 ferulic acid; 4coptisine; 5jatrorrhizine; 6 berberine; 7 hesperidin; 8 cinnamaldehyde; 9 aconitine; 10 ginsenoside Rb1; 11 6-gingerol.

The HPLC results are shown in Figures 1A–C. A total of eleven chemical components in WMW were determined, including citric acid, phellodendrine, ferulic acid, coptisine, jatrorrhizine, berberine, hesperidin, cinnamaldehyde, aconitine, ginsenoside Rb1, and 6-gingerol.

### Medicine Wumeiwan Significantly Alleviates Dextran Sulfate Sodium-Induced Acute Colitis in Mice

#### Medicine Wumeiwan Significantly Alleviates Dextran Sulfate Sodium-Induced Acute Colitis in Mice

**FIGURE 2:**
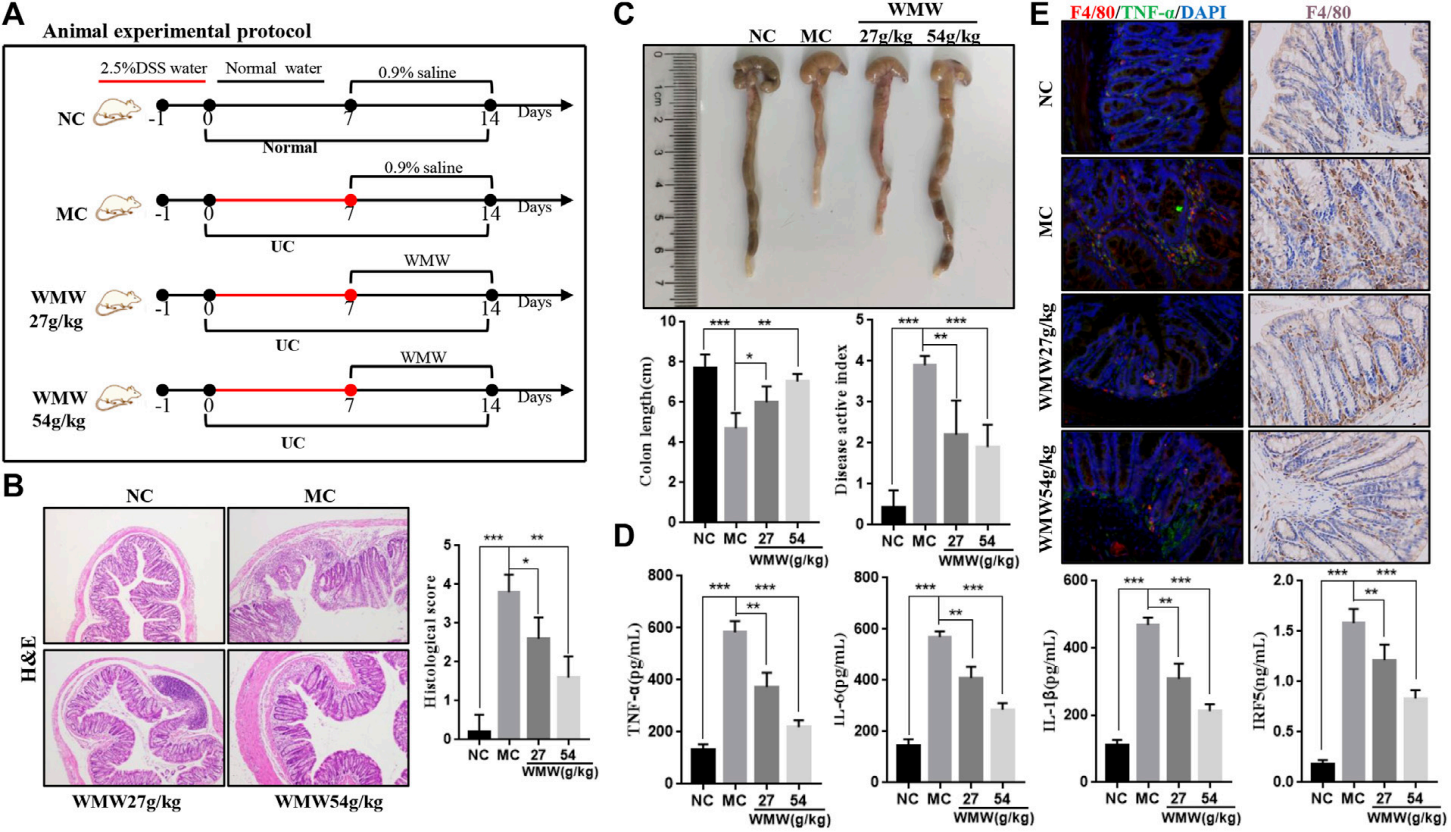
Effect of WMW on DSS-induced acute colitis in mice. **(A)** Scheme of experimental procedure for C57BL/6 mice were given drinking water containing 2.5% DSS for 1 week followed by oral administrated with WMW (27 g/kg, 54 g/kg) for 1 week. **(B)** Representative colon H&E staining and the histological score at different groups (original magnification, 100 ×). **(C)** Representative colon images and the length of different groups. DAI of mice was recorded weekly during the experiment. **(D)** Expression of TNF-*α*,IL-1, IL-6 and IRF5 in colon tissue were determined by ELISA from DSS-challenged mice **(E)** Double immunofluorescent signals from F4/80 and TNF-*α*, F4/80 Immunohistochemistry in DSS-induced colitis tissue at different groups (original magnification, 400 ×). *n* = 7–10, means ± SEM.*p < 0.05, **p < 0.01,***p < 0.001.

The results showed that WMW could significantly improve inflammatory cell infiltration and crypt injury in the colonic tissues of colitis mice, and it resulted in significantly decreased pathological scores (Figure 2B). We assessed DAI scores every week and measured colon length after treatment. The results indicated that WMW decreased the DAI scores of the colitis mice and that colon length was significantly greater after WMW treatment than in the model group (Figure 2C). Following that, we used an ELISA to measure the levels of inflammatory factors TNF-*α*, IL-1, IL-6, and IRF5 in colonic tissues. Overactivation of macrophages in inflammatory damage sites promotes the secretion of TNF-*α*, IL-1, and IL-6, while IRF5 promotes the recruitment of monocytes to colonic injury sites and conversion to activated macrophages (Corbin et al., 2020). Based on the results, WMW significantly decreased the levels of these inflammatory factors, and this effect became more significant as the dose increased (Figure 2D). The immunohistochemistry and double immunofluorescence results showed that the number of F4/80-positive macrophages significantly increased in the inflammatory damage sites. At the same time, F4/80 was found to be co-expressed with some TNF-*α*, proving that macrophage activation in inflammatory damage sites increased inflammation. After the WMW intervention, the co-expression of these two molecules decreased significantly; this effect was more significant with the 54 g/kg WMW dose (Figure 2E). These data therefore show that WMW could inhibit macrophage activation and decrease the secretion of inflammatory factors to alleviate DSS-induced acute colitis in mice and that a 54 g/kg dose has the best efficacy.

### Medicine Wumeiwan Significantly Alleviates Dextran Sulfate Sodium-Induced Chronic Colitis in Mice

**FIGURE 3:**
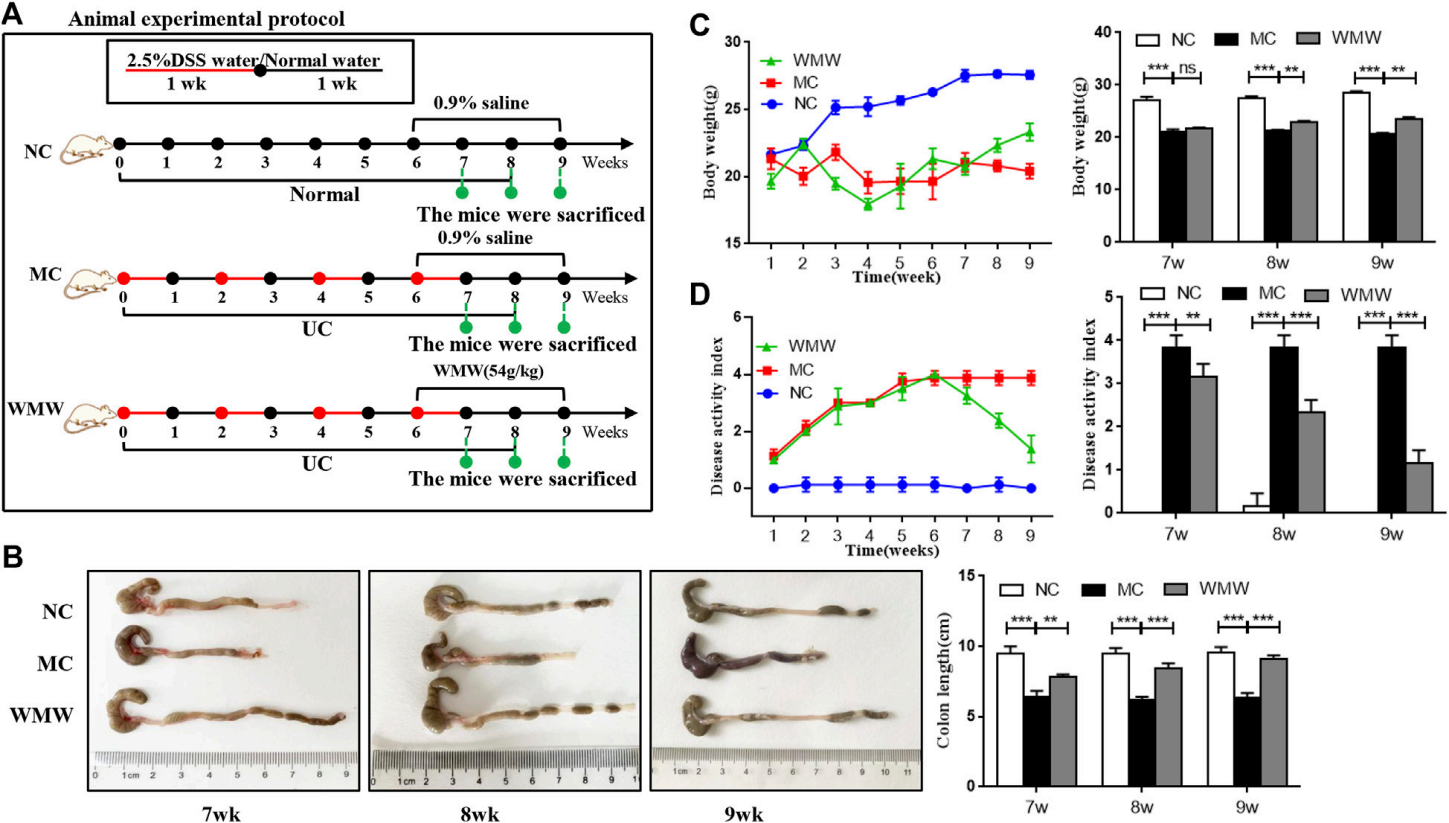
Effect of WMW on DSS-induced chronic colitis in mice. **(A)** Scheme of experimental procedure for C57BL/6 mice were given drinking water containing 2.5% DSS for 1 week followed by normal drinking water for 2 weeks, This step was repeated four times for 8 weeks Mice were oral administrated with WMW (54 g/kg) for 3 weeks, starting at 7 weeks post DSS challenge **(B)** Representative colon images and the length of different groups. **(C)** DAI of mice was recorded weekly during the experiment. **(D)** Body weight of mice was recorded daily during the experiment. *n* = 6–8, means ± SEM. *p < 0.05, **p < 0.01, ***p < 0.001.

To observe the effects of WMW on DSS-induced chronic UC in mice, mice were allowed *ad libitum* access to DSS for four cycles to construct a chronic UC model. At the end of the third cycle (week 6), gavage was carried out with a WMW dose of 54 g/kg. Mouse weight was measured and DAI scoring was performed once every week. In weeks 7, 8, and 9, colon length was measured and a statistical analysis of weight and DAI data was conducted (Figure 3A). As expected, compared with the model group, the results in the treatment group showed that WMW can prevent weight loss in colitis mice (Figure 3C) and decrease DAI (Figure 3D). As the dosing period increased, this effect became more significant. Similarly, the colon length of mice in the WMW group was significantly longer than in the model group (Figure 3B). This indicates that WMW can alleviate DSS-induced chronic UC in mice.

### Medicine Wumeiwan Can Decrease Macrophage Infiltration and Activation and Alleviate Colonic Tissue Damage

**FIGURE 4:**
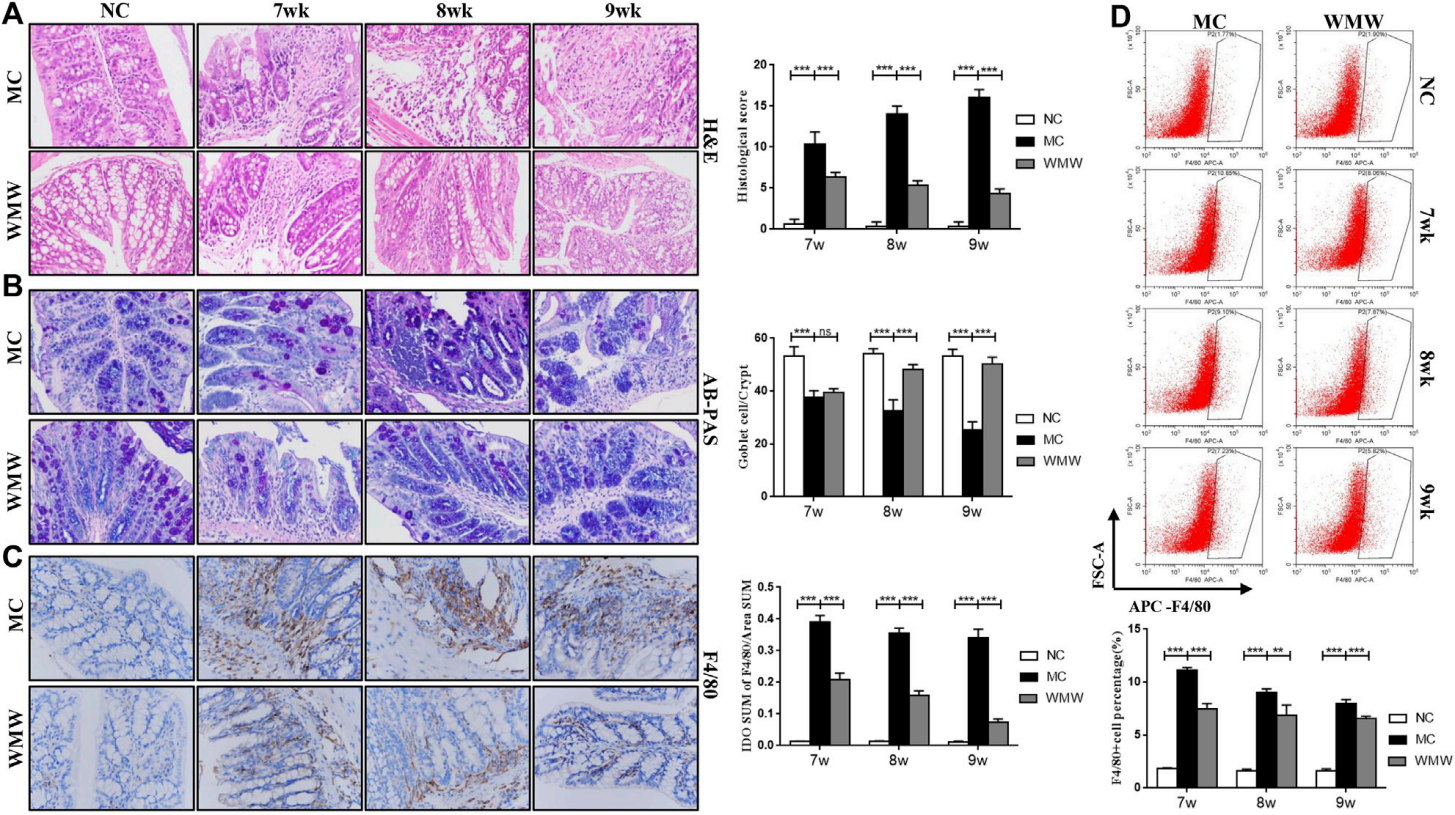
WMW significantly decrease macrophage infiltration and activation and alleviate colonic tissue damage. **(A)** Representative colon H&E staining and the histological score at different time points of different groups (original magnification, 200 ×). **(B)** Representative colon AB-PAS staining and the mean number of goblet cells at different time points of different groups (original magnification, ×200). **(C)** F4/80 Immunohistochemistry in DSS-induced colitis tissue at different groups (original magnification, 400 ×) **(D)** The proportion of macrophages in mice colon tissues at different time points was detected by flow cytometry. *n* = 3,means ± SEM.*p < 0.05, **p < 0.01, ***p < 0.001.

To observe the effects of WMW on colonic tissue damage in DSS-induced chronic colitis in mice, we carried out H&E and PAS staining of mouse colonic tissues and conducted histopathological scoring of the results. The mean number of goblet cells in every crypt was enumerated. Immunohistochemistry and flow cytometry were used to measure macrophage infiltration and counts in inflammation sites. The H&E staining analysis results showed that colitis mice developed typical inflammatory cell infiltration and crypt damage. The aforementioned pathological presentations in the WMW group significantly improved, and the histopathological score of the WMW group was lower than that of the model group (Figure 4A). PAS staining analysis showed that the goblet cell count in the colons of colitis mice significantly decreased and that mucosal secretion decreased. In the WMW group, goblet cell count significantly increased, and mucosal secretion also increased (Figure 4B). The immunohistochemical results showed extensive infiltration of F4/80-positive macrophages in the colonic mucosa and infiltration even in the muscular layer. The flow cytometry results further proved that the number of activated macrophages was significantly increased in inflammatory damage sites. After WMW treatment, the number of F4/80-positive macrophages in inflammatory damage sites gradually decreased, and the degree of infiltration gradually decreased (Figures 4C,D). These changes became more significant as the treatment duration increased. These data show that WMW could alleviate intestinal inflammation and tissue damage in DSS-induced colitis mice.

### Medicine Wumeiwan Decreases Colonic and Serum Pro-inflammatory Factor Levels and Increases Anti-inflammatory Factor Levels in Dextran Sulfate Sodium-Induced Colitis

**FIGURE 5:**
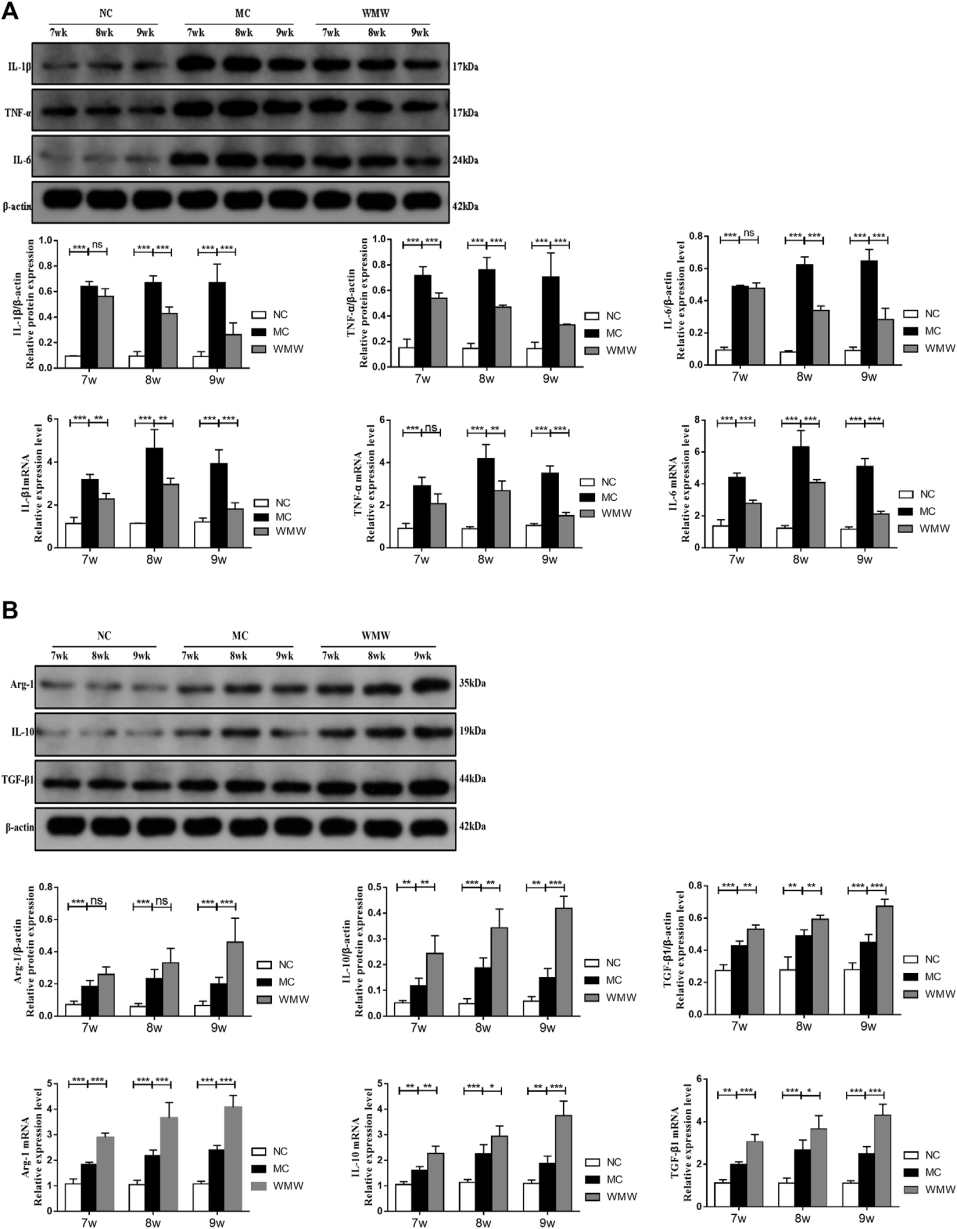
WMW reduces colonic inflammatory responses in DSS-induced colitis. **(A)** Relative mRNA level of IL-1*β*, IL-6, and TNF-*α* in the colon was measured through qPCR. Expression of IL-1*β*, IL-6, and TNF-*α* in colon tissue lysates were determined by Western blot from DSS-challenged mice. **(B)** Relative mRNA level of IL-10, Arg-1, and TGF-*β*1in the colon was measured through qPCR. Expression of IL-10, Arg-1, and TGF-*β*1 in colon tissue lysates were determined by Western blot from DSS-challenged mice. *n* = 3,.means ± SEM.*p < 0.05, **p < 0.01, ***p < 0.001.

To observe the effects of WMW on intestinal inflammation in DSS-induced colitis mice, we utilized quantitative reverse transcription-polymerase chain reaction (qRT-PCR) and Western blot to measure the levels of pro-inflammatory factors TNF-*α*, IL-1*β*, and IL-6 and anti-inflammatory factors IL-10, Arg-1, and TGF-*β*1 in serum and colon tissues. The results showed that, compared with the control group, the levels and expression of pro-inflammatory factors TNF-*α*, IL-1*β*, and IL-6 in colon tissues from colitis mice were significantly increased. Compared with the model group, the WMW treatment group had significantly decreased levels and expression of these factors (Figure 5A). Compared with the control group, the levels and expression of anti-inflammatory factors IL-10, Arg-1, and TGF-*β*1 in colon tissues were increased in the treatment group. This finding is consistent with the results reported in the literature showing that the release of considerable amounts of pro-inflammatory factors worsens inflammation. However, the body also secretes anti-inflammatory factors to alleviate the damage caused by these pro-inflammatory factors (Cao et al., 2019). After WMW treatment, the levels and expression of IL-10, Arg-1, and TGF-*β*1 were further increased. These effects became more significant as the treatment duration increased (Figure 5B). Thus, WMW can inhibit the secretion of pro-inflammatory factors and promote the secretion of anti-inflammatory factors. Inflammatory factors are intimately associated with UC pathogenesis, in which macrophages are the major immune cells secreting inflammatory factors. Macrophages can be a double-edged sword, depending on their phenotype, as they can secrete pro-inflammatory factors that aggravate inflammation but also anti-inflammatory factors that alleviate inflammation (Locati et al., 2020). This result shows that WMW participates in the regulation of macrophage activation and phenotype.

### Effects of Medicine Wumeiwan on Macrophage M1/M2 Polarization and Expression of Inflammatory Factors in Colonic Tissues in Dextran Sulfate Sodium-Induced Colitis Mice

**FIGURE 6:**
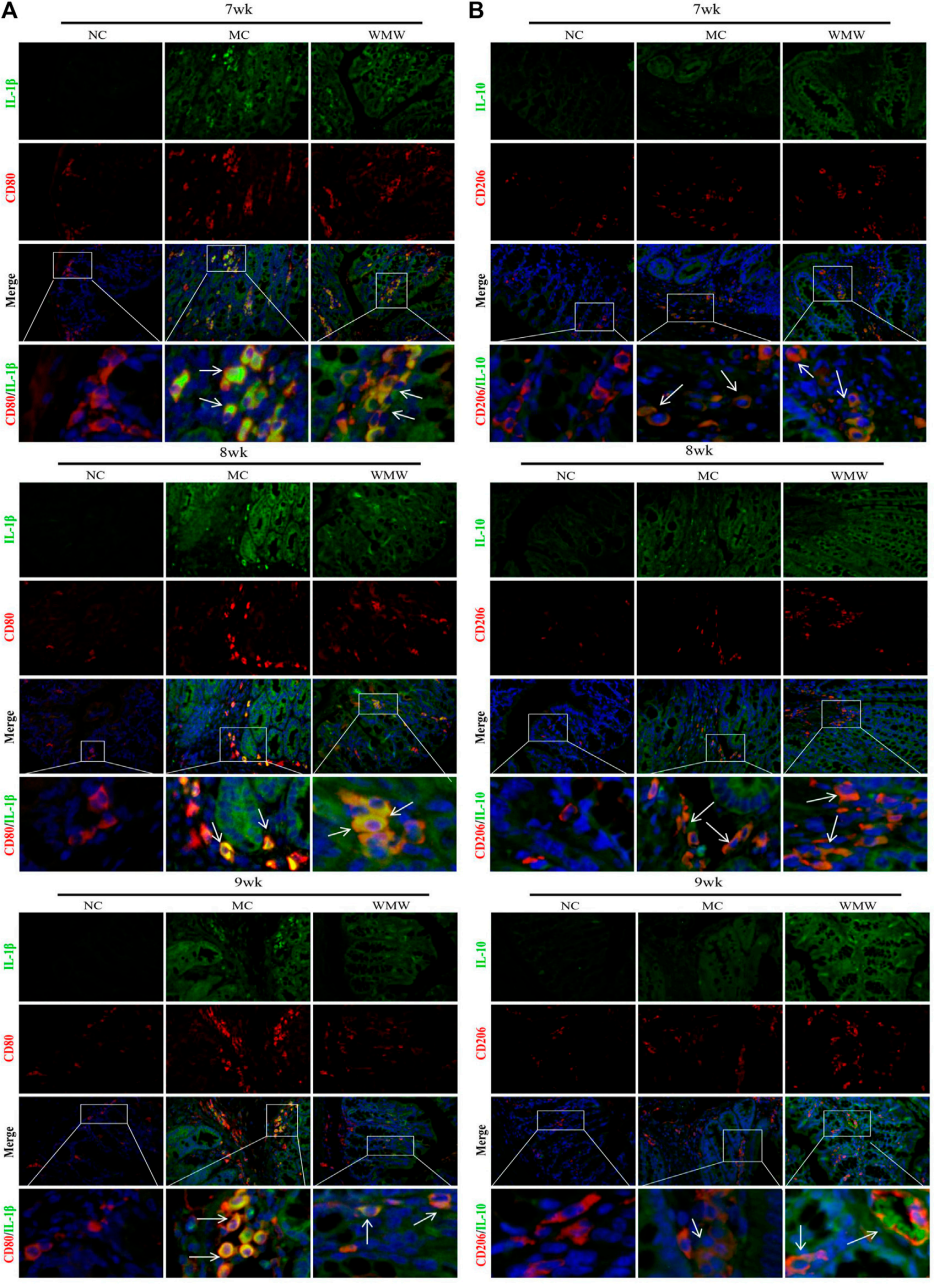
The proportion of M1/M2 macrophages changes in colon sections obtained from mice with ulcerative colitis induced by DSS **(A)** Double immunofluorescent signals from CD80 and interleukin-1 in DSS-induced colitis tissue at different time points (original magnification, 200 ×). **(B)** Double immunofluorescent signals from CD206 and interleukin-10 in DSS-induced colitis tissue at different time points (original magnification, 200 ×).

To study whether the effects of WMW on inflammatory factors are related to macrophage M1/M2 polarization, we employed double immunofluorescence to observe the co-expression of M1 macrophages and related pro-inflammatory factor IL-1*β* and of M2 macrophages and anti-inflammatory factor IL-10. CD80 was used as a marker for M1-positive macrophages, and CD206 was used as a marker for M2-positive macrophages. The CD80 and IL-1*β* double immunofluorescence results showed that, in the control group, there was minimal CD80 expression, and IL-1*β* was not expressed. In the model group, CD80 and IL-1 co-expression was significantly increased. Compared with the model group, CD80 and IL-1*β* co-expression was gradually reduced in the WMW group in direct proportion to the treatment duration (Figure 6A). This result shows that the pro-inflammatory factor IL-1*β* is secreted by M1-polarized macrophages and that WMW could inhibit M1 macrophage polarization and secretion of the pro-inflammatory factor IL-1*β*. In the control group, the CD206 and IL-10 double immunofluorescence results showed that there was minimal CD206 expression, and IL-10 was not expressed. In the model group, CD206 and IL-10 co-expression increased. Compared with the model group, CD206 and IL-10 co-expression was further increased in the WMW group in direct proportion to the treatment duration (Figure 6B). This result shows that the anti-inflammatory factor IL-10 is secreted by M2-polarized macrophages and that WMW could promote M2 macrophage polarization and secretion of the anti-inflammatory factor IL-10. Hence, WMW can promote the conversion of colonic M1 macrophages to M2 in DSS-induced colitis mice and simultaneously inhibit the activation of pro-inflammatory factors and promote the activation of anti-inflammatory factors.

### Medicine Wumeiwan Can Decrease the Percentage of M1 Macrophages and Increase the Percentage of M2 Macrophages in Colonic Tissues in Dextran Sulfate Sodium-Induced Mouse Colitis

**FIGURE 7:**
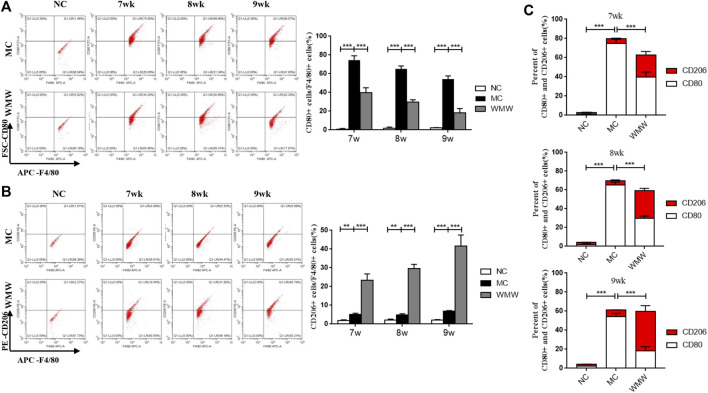
WMW Impact the M1 versus M2 Polarization of Macrophages in mice with ulcerative colitis induced by DSS **(A)** The proportion of M1 macrophages in mice colon tissues at different time points was detected by flow cytometry. **(B)** The proportion of M2 macrophages in mice colon tissues at different time points was detected by flow cytometry. **(C)** Ratio of M1/M2 macrophages changes at different time points in the different groups. *n* = 3, means ± SEM.*p < 0.05, **p < 0.01, ***p < 0.001.

To further study the effects of WMW on the M1/M2 macrophage ratio in colonic tissues from DSS-induced mouse colitis, we employed flow cytometry to measure the proportions of M1 and M2 macrophages. F4/80 was used as a macrophage marker, CD80 was used as a marker for M1-positive macrophages, and CD206 was used as a marker for M2-positive macrophages. The results showed that, compared with the control group, the percentage of CD80-labeled M1-positive macrophages was significantly increased in colonic tissues from the colitis mouse group, reaching a peak at week 7 and persisting until week 9. The percentage of CD206-labeled M2-positive macrophages significantly increased. After WMW treatment, the percentage of CD80-labeled M1-positive macrophages gradually decreased and was inversely proportional to the treatment duration (Figure 7A), while the percentage of CD206-labeled M2-positive macrophages further increased in direct proportion to the treatment duration (Figure 7B). To intuitively reflect the inhibition of macrophage M1 polarization and promotion of M2 polarization by WMW through the reduction of colonic and serum pro-inflammatory factor levels and increase of anti-inflammatory factor levels, we calculated the M1/M2 macrophage ratio at different time points. The proportion of M1 macrophages was far higher than that of M2 macrophages in the model group. After WMW treatment, the ratio of M2 macrophages gradually increased, while that of M1 macrophages gradually decreased as the treatment duration increased (Figure 7C). These data further prove that WMW could inhibit M1 polarization and promote M2 polarization in colonic macrophages in DSS-induced colitis mice.

### Network Pharmacology Prediction of Medicine Wumeiwan Treatment of Ulcerative Colitis

**FIGURE 8:**
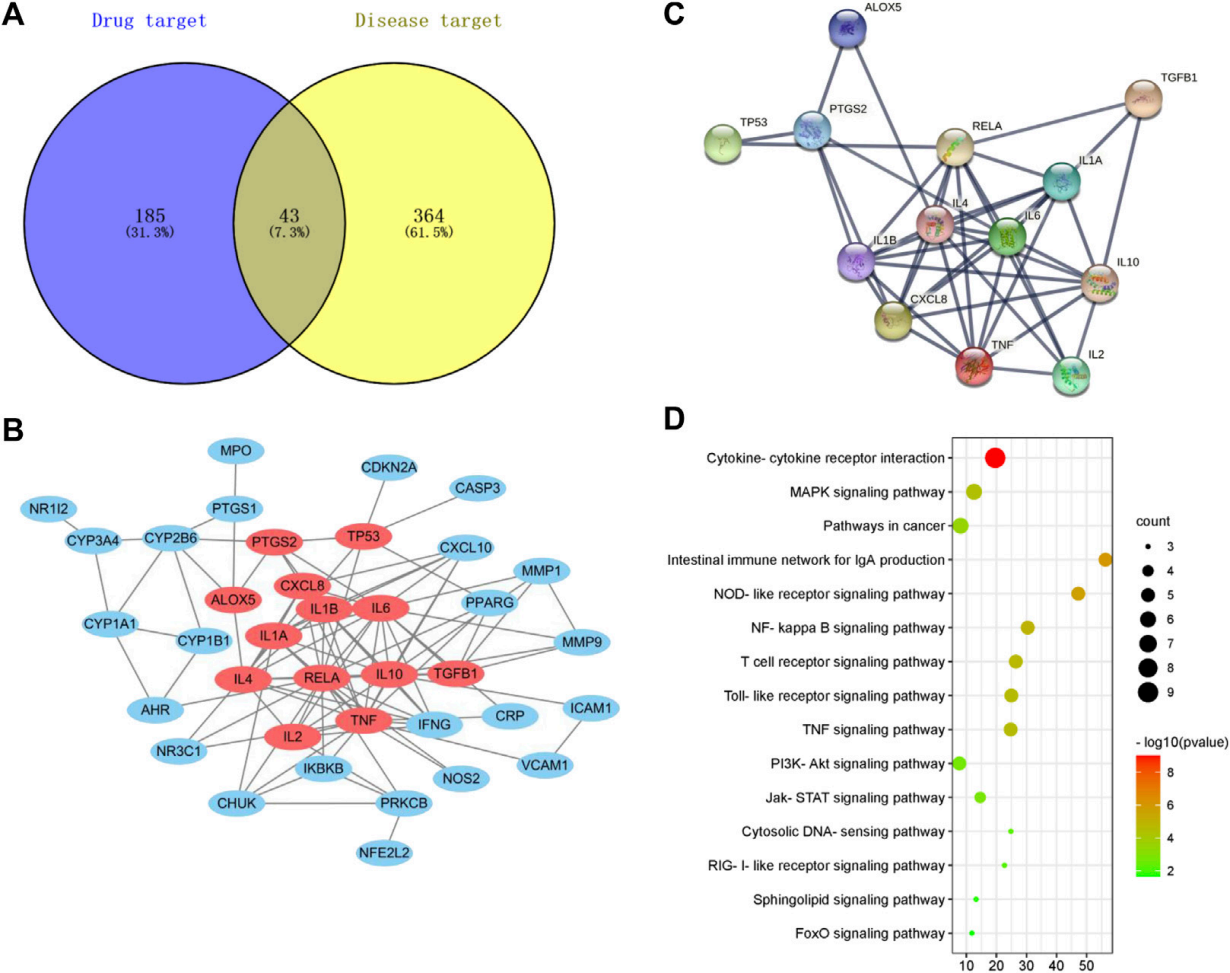
Network pharmacology prediction of WMW treatment for DSS-induced **colitis**. **(A)** The intersection of WMW targets and disease targets. **(B)** 43 core targets of WMW acting on DSS-induced **colitis**. **(C)** The result of PPI analysis for potential targets of WMW. **(D)** The result of KEGG enrichment analysis for the potentials pathway of WMW.

To examine the therapeutic effector mechanism of WMW in UC, we employed network pharmacology to analyze the potential effector targets of WMW in UC. The results showed that there were 43 cross-targets between WMW and UC (Figure 8A). Protein–protein interaction (PPI) network analysis of the 43 cross-targets found that WMW acts on 13 core target genes in DSS-induced UC (Figures 8B,C). Kyoto Encyclopedia of Genes and Genomes (KEGG) pathway enrichment analysis results showed that nine core target genes were enriched in cytokine and cytokine receptor interactions, six target genes were enriched in the mitogen-activated protein kinase (MAPK) pathway, and five target genes were enriched in the NF-κB pathway (Figure 8D). The results showed that WMW may treat DSS-induced UC through multiple pathways and multiple targets and that the MAPK and NF-κB pathways may be intimately associated with DSS-induced UC.

### Medicine Wumeiwan Inhibition of Macrophage M1 Polarization and Promotion of M2 Polarization May Be Associated With the p38 MAPK, NF-κB, and STAT6 Signaling Pathways

**FIGURE 9:**
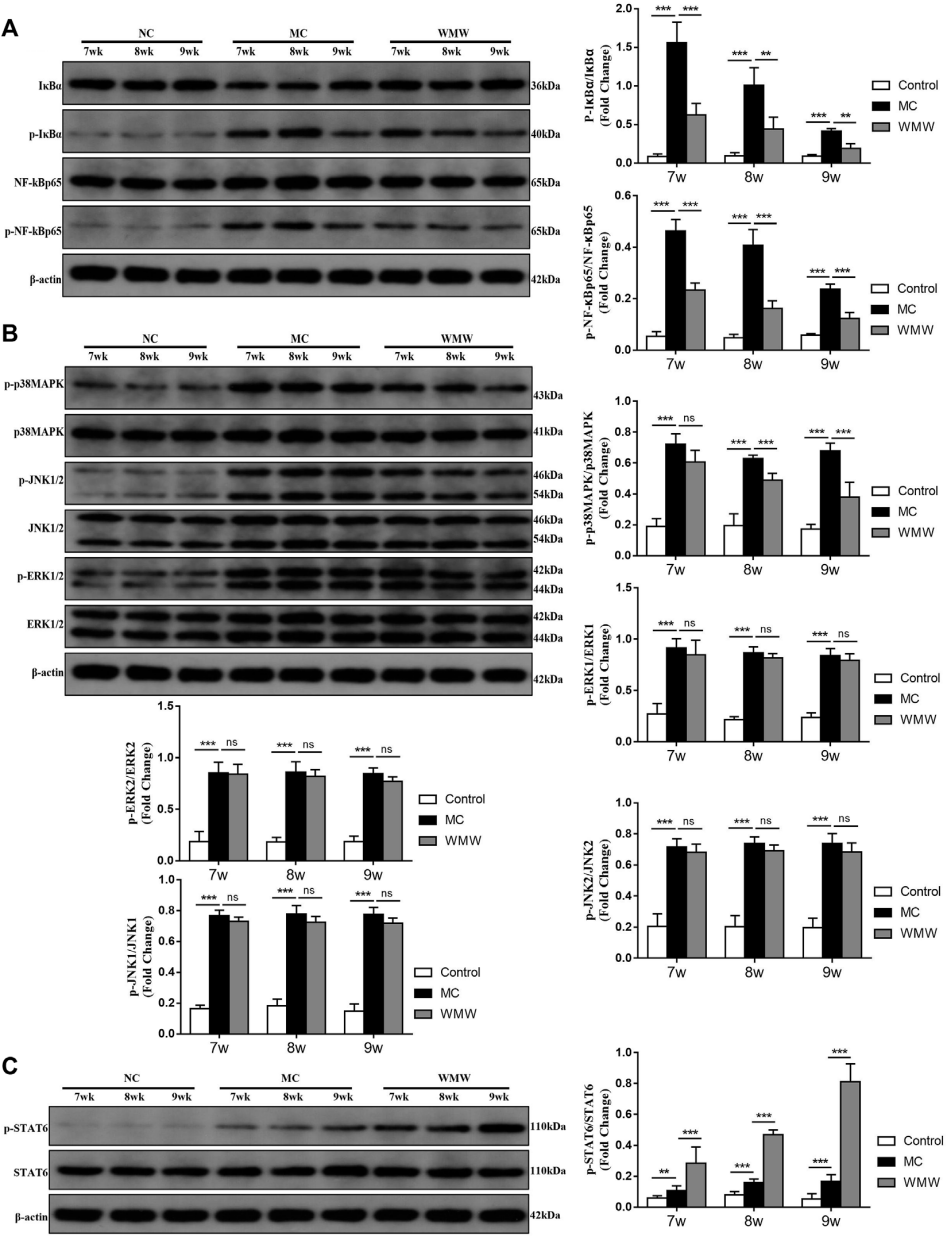
The effects of WMW on the p38 MAPK, NF-κB, and STAT6 signaling pathways after ulcerative colitis. **(A)** Expression of MAPK signaling pathway related proteins P38 MAPK, ERK1/2 and JNK1/2 in colon tissue lysates were determined by Western blot from DSS-challenged mice **(B)** Expression of NF-κB signaling pathway related proteins NF- κBp65,IκB*α* in colon tissue lysates were determined by Western blot from DSS-challenged mice. **(C)** Expression of STAT6, and p-STAT6 in colon tissue lysates were determined by Western blot from DSS-challenged mice. *n* = 3, means ± SEM.*p < 0.05, **p < 0.01, ***p < 0.001.

Based on the network pharmacology analysis results, we further verified changes in the MAPK and NF-κB pathways and core target-related pathways in DSS-induced UC. The results showed that MAPK and NF-κB are core pathways in macrophage M1 polarization, while STAT6 participates in macrophage M2 polarization (Murray, 2017). We employed Western blotting to measure changes in the protein levels of P38MAPK, jun N-terminal kinase (JNK), and extracellular signal-regulated kinase (ERK) in the MAPK signaling pathway and related proteins in the NF-κB and STAT6 signaling pathways. The Western blotting results showed that NF-κBp65, P38 MAPK, ERK1/2, and JNK1/2 expression and phosphorylation were significantly increased in the model group, IκB*α* protein expression was significantly decreased and phosphorylation significantly increased (Figures 9A,B), and STAT6 protein expression and phosphorylation were increased (Figure 9C). These changes were directly proportional to the model construction duration. After WMW intervention, the expression and phosphorylation of NF-κBp65 and P38 MAPK gradually decreased with the treatment duration, while IκB*α* protein expression gradually increased and phosphorylated IκB*α* gradually decreased. STAT6 protein expression and phosphorylation also significantly increased, but there were no significant changes in ERK1/2 or JNK1/2 expression and phosphorylation. This shows that DSS gradually activates the P38MAPK, JNK, ERK, and NF-κB signaling pathways and that it also activates the STAT6 signaling pathway but to a lesser degree. WMW gradually inhibits the activation of the P38MAPK and NF-κB signaling pathways and further activates the STAT6 signaling pathway.

## Discussion

WMW, a traditional medicine in China, has been used to treat chronic intestinal inflammatory disorders for two millennia. Recent studies have found that WMW significantly reduces the acute colonic inflammatory response induced by trinitrobenzenesulfonic acid by reducing the infiltration of macrophages and neutrophils and the levels of local IL-1*β*, IL-6, TNF-*α*, and IFN-*γ* (Wu F. et al., 2021). UC is a chronic inflammatory disorder, and DSS is commonly used to induce UC in animal models. The histopathological changes in UC development in animals are similar to those in human UC, so this animal model is commonly used to evaluate drug efficacy (Li et al., 2018). Therefore, *ad libitum* access to DSS (2.5% w/v) for one and four cycles was used in this study to induce acute and chronic UC, respectively, to further understand the effector mechanisms of WMW in UC treatment.

UC is characterized by chronic inflammation of the intestinal mucosa, and its pathogenesis involves many factors, such as immunity, genetics, and the environment (Meroni et al., 2019). Macrophages are among the most abundant cells in the intestinal mucosa. The imbalance of intestinal macrophage polarization, that is, the abnormal response of bacteria and bacterial antigens, triggers and drives the excessive inflammatory immune response; this imbalance is considered to be one pathogenesis of UC (Sun et al., 2020). In a healthy intestinal environment, intestinal macrophages exhibit an anti-inflammatory, tolerant M2 phenotype. However, during inflammation, monocytes are recruited into the lamina propria of the intestine and become inflammatory macrophages, secrete pro-inflammatory cytokines, and promote the inflammatory response. In this study, a large number of M1 macrophages was detected in UC patients and in the UC model, but some M2 macrophages were also present. Although no quantitative analysis of these two macrophage phenotypes has been carried out, these M2 macrophages still have anti-inflammatory effects and promote healing (Isidro and Appleyard, 2016). Macrophage polarization is intimately associated with UC progression and is considered a new target for therapeutic intervention. Our study results show that macrophage activation and infiltration are significantly increased in injury sites in DSS-induced mouse colitis. The double immunofluorescence and flow cytometry results show that M1/M2 macrophage expression and relative numbers were significantly higher than in the control group. However, the expression and number of M1 macrophages were significantly higher than M2 macrophages, which is consistent with the results reported in the literature (Wu MM. et al., 2021). After WMW treatment, M1 macrophage expression and relative counts decreased in colon injury sites, while M2 macrophages significantly increased and changed in these two types of macrophages, becoming more significant as the treatment duration increased. This result shows that WMW can interfere with macrophage M1/M2 polarization, thereby affecting inflammatory responses.

Inflammatory factors are the key to the cell activity of macrophages. Under different polarization states, macrophages secrete different inflammatory factors to exert their immune effects. M1 macrophages secrete pro-inflammatory factors, such as TNF-*α*, IL-6, and IL-1, thus accelerating the inflammation process and damaging body tissues. TNF-*α*, IL-6, and IL-1 not only exacerbate colon inflammation but are also considered to be indicators of disease severity (Haberman et al., 2019). M2 macrophages secrete anti-inflammatory factors, such as IL-10, ARG-1, and TGF-*β*1, slowing down the process of inflammation and repairing body tissues. These anti-inflammatory factors not only inhibit the secretion of pro-inflammatory factors to reduce inflammation damage but also participate in the repair of damaged mucosa (Formentini et al., 2017). Therefore, we measured the levels of M1 macrophage-related inflammatory factors (TNF-*α*, IL-6, and IL-1) and M2 macrophage-related inflammatory factors (IL-10, ARG-1, and TGF-*β*1) in serum and colon tissues. Our study results proved that the levels and expression of pro-inflammatory factors (TNF-*α*, IL-6, and IL-1*β*) and anti-inflammatory factors (IL-10, Arg-1, and TGF-*β*1) significantly increased in serum and colonic tissues after colitis was induced by DSS in mice, which is content with the changes in M1/M2 macrophage expression and relative M1/M2 ratios. After WMW treatment, the levels and expression of pro-inflammatory factors (TNF-*α*, IL-6, and IL-1*β*) significantly decreased, while the levels and expression of anti-inflammatory factors (IL-10, Arg-1, and TGF-*β*1) further increased. Studies have proven that IL-10 limits excessive inflammation, upregulates innate immunity, promotes tissue repair, and maintains tissue homeostasis during inflammation (Ouyang and O'Garra, 2019). Although TGF-*β*1 is considered to be a cause of UC aggravation, the main effector mechanism of TGF-*β*1 is the activation of the downstream Smad signaling pathway to promote fibroblast activation (Naghdalipour et al., 2020). This study found that an increase in serum and tissue TGF-*β*1 is consistent with UC alleviation, consistent with the results reported in the literature (Xu et al., 2019). Argininase-1 (Arg-1) competes with inducible nitric oxide synthase for L-arginine to decrease nitric oxide (NO) synthesis, which alleviates inflammation (Aratake et al., 2018). This result further shows that WMW regulates macrophage M1/M2 polarization, decreases the secretion of pro-inflammatory factors (TNF-*α*, IL-6, and IL-1), promotes the secretion of anti-inflammatory factors (IL-10, Arg-1, and TGF-*β*1), and inhibits intestinal inflammatory responses to treat UC.

WMW can regulate macrophage M1/M2 polarization in colonic tissues and inhibit inflammatory responses, but the specific mechanism by which it interferes with macrophage M1/M2 polarization remains unclear, and there is a dearth of literature on this question. Currently, network pharmacology is widely used in traditional Chinese medicine studies and has significant strengths in revealing the potential targets and effector mechanisms of drugs (Zhang et al., 2019). In this study, network pharmacology was used to predict the targets and pathways relevant to UC treatment by WMW. The results show that the targets of UC treatment by WMW are complex and associated with many signaling pathways, such as the MAPK, NF-κB, and JAK-STAT pathways, of which JAK-STAT, NF-κB, and MAPK participate in macrophage polarization and functional regulation (Arya et al., 2018; Rahal et al., 2018; Dorrington and Fraser, 2019). There are four members of the MAPK family: ERK,JNK,P38MAPK, and ERK5. ERK is ubiquitous in many tissues and participates in regulating cell proliferation and differentiation. The JNK family is composed of crucial signal transduction molecules induced by various stresses in cells, and they participate in cellular responses to radiation, osmotic pressure, and temperature changes. p38MAPK mediates inflammation and apoptosis and is used as a target for the development of anti-inflammatory drugs (Dorrington and Fraser, 2019). NF-κB is an internal signaling pathway that participates in macrophage M1 polarization and is a “start pathway” in immune function in macrophages. Its activation is regulated by IκB. In the quiescent state, IκB and NF-κB form a heterodimer that is localized to the cytoplasm and inhibits NF-κB activation. When inflammatory responses are activated, IκB and NF-κB dissociate, IκB is gradually phosphorylated (p-IκB), and the inhibitory effect of IκB on NF-κB gradually decreases. NF-κB is phosphorylated and enters the nucleus to carry out its biological activity of activating and secreting downstream inflammatory factors, and inflammatory responses are gradually amplified (Zhong et al., 2016). Meanwhile, STAT6 mediates macrophage M2 polarization (Yu et al., 2019).

Therefore, by combining network pharmacology with results reported in the literature, we were able to further study changes in the P38MAPK, ERK, JNK, NF-κB, and STAT6 signaling pathways, which are intimately associated with macrophage polarization in DSS-induced colitis. Our study results prove that phosphorylated P38MAPK, JNK, and ERK in the MAPK pathway were significantly upregulated in DSS-induced mouse colitis and that the expression of phosphorylated IκB in the NF-κB signaling pathway was increased. IκB protein expression was decreased in the model, which decreases the inhibitory effects on NF-κB, and NF-κBp65 was gradually phosphorylated, ultimately upregulating the NF-κB signaling pathway. The expression of phosphorylated STAT6 protein was also gradually increased, suggesting that the STAT6 signaling pathway was activated. After WMW treatment, activation of the P38MAPK and NF-*β* signaling pathways was significantly downregulated, no changes occurred in the JNK and ERK signaling pathways, and the related STAT6 signaling pathway was further upregulated. These changes were directly proportional to the treatment duration, whereas the duration had no significant effect on the JNK and ERK signaling pathways.

**FIGURE 10:**
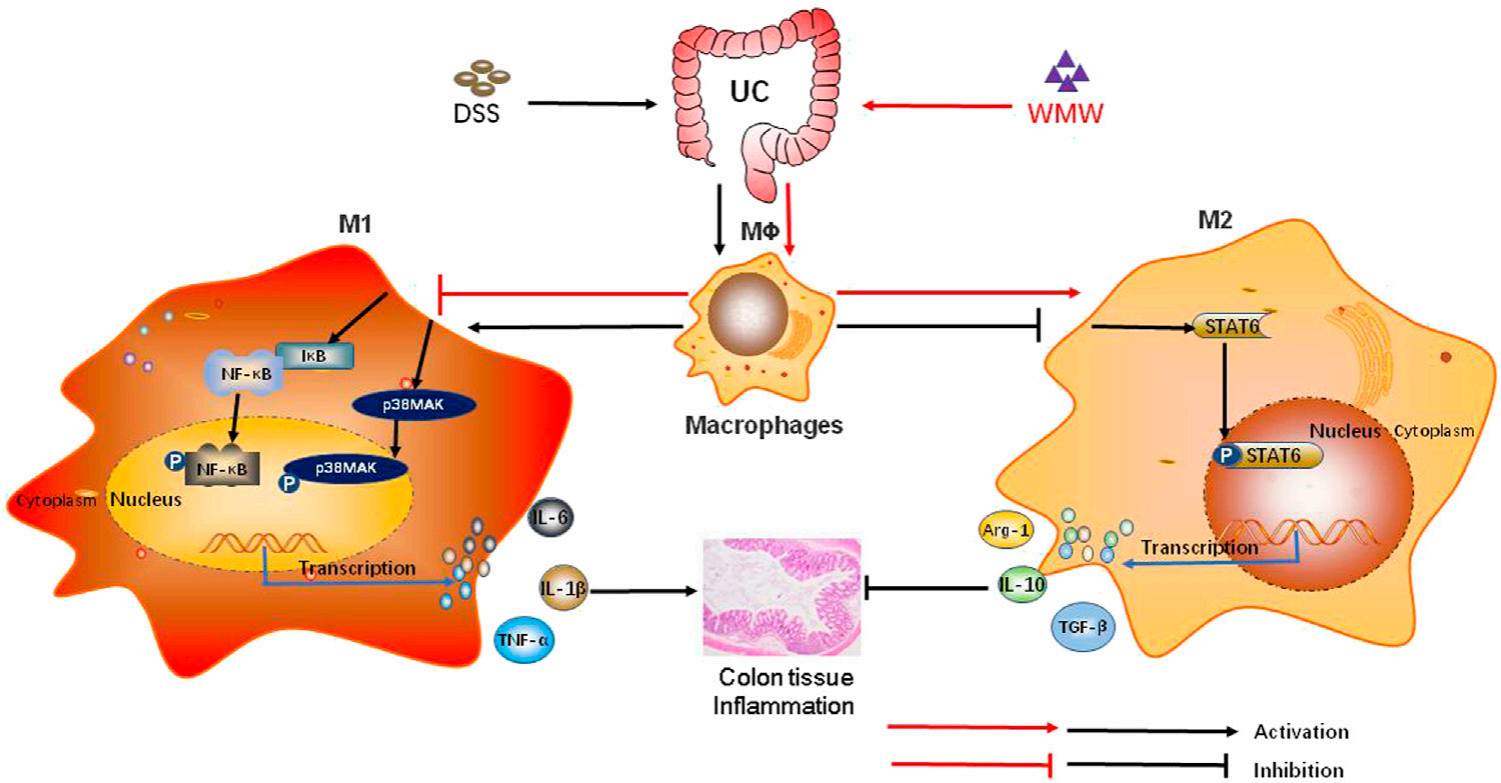
The potential role of WMW in DSS-induced colitis in mice. WMW treatment can Inhibits macrophage activation, Meanwhile regulate colonic macrophages M1/M2 polarization, that is,Inhibits M1 polarization and reduces the pro-inflammatory cytokines IL-1*β*, IL-6, TNF-*α* activation and secretion, enhance M2 activity. polarization and increase the anti-inflammatory cytokines IL-10, ARG-1, TGF-*β*1 activation and secretion. WMW gradually inhibits the activation of the P38MAPK and NF-κB signaling pathways and further activates the STAT6 signaling pathway.

In conclusion, our data show that WMW interferes with macrophage M1/M2 polarization to ameliorate colitis. Its protective mechanisms are associated with the activation of the P38MAPK and NF-κB signaling pathways and promoting the activation of the STAT6 signaling pathway, showing that WMW has dual regulatory mechanisms in macrophage M1/M2 polarization (Figure 10). *In vitro* cellular studies have shown that the active ingredient of WMW, berberine, can decrease the secretion of mature interleukin (IL)-1*β* in LPS-stimulated RAW264.7 macrophages (Wu et al., 2019). In addition, berberine can inhibit and significantly decrease the activation of LPS-stimulated RAW264.7 macrophages, and it limits the secretion of inflammatory factors and chemokines (Vivoli et al., 2016). Ginsenoside Rbl can promote the expression of Arg-1 and macrophage mannose receptor (CD206), two classical M2 macrophage markers, in primary peritoneal mouse macrophages, increase serum IL-4 and IL-13 secretion, and promote STAT6 phosphorylation (Zhang et al., 2018). Cinnamaldehyde inhibits MAPK phosphorylation and pro-inflammatory factor expression to produce anti-inflammatory effects on LPS-stimulated RAW264.7 macrophages (Kim et al., 2018). Ferulic acid and its complex significantly inhibit I-kappa B degradation in LPS-stimulated RAW264.7 macrophages and decrease the release of the inflammatory factor TNF-*α* (Murakami et al., 2002). Citric acid-treated cereal germ could specifically inhibit the phosphorylation of NF-κB p65 and p38 in peritoneal macrophages from BALB/c mice and decrease the production of inflammatory factors (TNF*α* and IL-6) and COX-2 (Jeong et al., 2017). The synergistic effects of these active ingredients *in vivo* may be the material basis of how WMW regulates macrophage M1/M2 polarization. Our study is the first to reveal the effects and mechanisms of WMW treatment of UC from the perspective of macrophage M1/M2 polarization, and the results provide theoretical support for the clinical application of WMW. This method provides new ideas and assistance for the secondary development, optimization, and innovation of traditional Chinese medicine prescriptions, solves the problem of previously unclear effector mechanisms in compound traditional Chinese medicine, and overcomes existing research and application limitations.

Although WMW is a promising anti-UC drug, this study has some limitations. First, we proved that WMW interferes with macrophage M1/M2 polarization to inhibit intestinal inflammation in *in vivo* animal experiments, but *ex vivo* cellular experiments are still required for further validation. However, WMW consists of many drugs and is administered orally, which poses a challenge for *ex vivo* experiments. Second, macrophage M1/M2 polarization participates in tissue repair in addition to inflammatory responses. Further studies are required to determine whether WMW can promote repair in damaged mucosal tissues. In addition, many studies have proven that WMW has a positive effect on UC, but there is currently no drug that can interfere with macrophage M1/M2 polarization. Therefore, there was no suitable positive control drug for this study. Finally, many signaling pathways participate in macrophage M1/M2 polarization, but our study validated only the effector mechanisms of WMW in the MAPK, NF-κB, and STAT6 signaling pathways. Hence, the mechanisms revealed were limited.

## Data Availability

The original contributions presented in the study are included in the article/Supplementary Material, further inquiries can be directed to the corresponding author.

## Glossary

Arg-1: arginase-1
CAC: colitis-associated colorectal cancer
DAI: Disease activity index
DSS: dextran sulfate sodium Salt
ELISA: enzyme-linked immunosorbent assay
ERK: extracellular regulated proteinhnase
H&E: hematoxylin-eosin
IκB: inhibitor kappa B alpha
IL-1: Interleukin-1
IL-4: Interleukin-4
IL-6: Interleukin-6
IL-10: Interleukin-10
IL-13: Interleukin-13
IRF5: Interferon regulatory factor 5
JNK: c-jun N-terminal kinase
LPS: lipopolysaccharide
MAPK: mitogen-activated protein kinases
M1: classically activated macrophages
M2: alternatively activated macrophages
NF-κB: nuclear factor-kappa B
PAS: periodic acid Schiff stain
p-Erk1/2: phosphorylated extracellular regulated proteinhnase1/2
p38MAPK: p38mitogen-activated protein kinases
p-p38MAPK: phosphorylated p38mitogen-activated protein kinases
p-IkB*α*: phosphorylated inhibitor kappa B alpha
p-NF-kBp65: phosphorylated nuclear factor-kappa Bp65
p-JNK1/2: phosphorylated c-jun N-terminal kinase1/2
p-STAT-6: phosphorylated signal transducers and activators of transcription-6
STAT: signal transducers and activators of transcription
TCM: traditional Chinese medicine
TGF-*β*1: Transforming growth factor-*β*1
TNBS: 2,4,6-trinitrobenzene sulfonic acid
TNF-*α*: Tumor necrosis factor-*α*
UC: Ulcerative colitis
WMW: Wu-Mei-Wan
